# Vertebral column decancellation in Pott’s deformity: use of Surgimap Spine for preoperative surgical planning, retrospective review of 18 patients

**DOI:** 10.1186/s12891-018-1929-6

**Published:** 2018-01-15

**Authors:** Wenhao Hu, Xuesong Zhang, Jiayi Yu, Fanqi Hu, Hao Zhang, Yan Wang

**Affiliations:** 1 The Department of Orthopedics, Chinese PLA General Hospital, Fuxing Rd. 28, Haidian District, Beijing, China; 2 Department of Renal cancer and Melanoma, Peking University Cancer Hospital, Fucheng Rd. 28, Haidian District, Beijing, China

**Keywords:** Pott’s deformity, Vertebral column decancellation, Surgimap Spine, Vertebral column resection, Pedicle subtraction osteotomy, Sagittal vertical angle, Visual analog scale, American spinal injury association

## Abstract

**Background:**

In the late stage of Spinal tuberculosis, the bony destruction and vertebral collapse often leads to significant kyphosis, presenting clinically as a painful gibbus deformity, with increased instability, vertebral body translations and increased risk of neurologic involvement. Vertebral column decancellation is thought to be suitable for most patients with severe rigid kyphosis. Surgimap Spine, could offer a pragmatic graphical method for the surgical planning of osteotomies. The aim of this study was to evaluate the efficacy of Vertebral column decancellation planned preoperatively with the computer software-assistance in the patients with Pott’s kyphosis.

**Methods:**

Between May 2012 and May 2015, 18 patients with Pott’s kyphosis underwent the Vertebral column decancellation using Surgimap Spine for preoperative surgical planning. Preoperative and postoperative Konstam’s angle, sagittal vertical angle, lumbar lordosis, thoracic kyphosis, pelvic tilt and pelvic incidence were measured. Visual analog scale and American Spinal Injury Association were documented.

**Results:**

The Konstam’s angles decreased from 88.1° (range, 70–105°) preoperatively to 18.5° (range, 7–31°) (*P* < 0.01). All patients reached the physiological limits at the final follow-up. The mean VAS score was reduced from preoperative 7.1 (range, 6–8) to 1.8 (range, 1–3, *P* < 0.01) and the ODI improved from 65.8% (range, 58–74%) to 20.2% (range, 12–38%, *P* < 0.01). At final follow-up, there was radiographic evidence of solid fusion at the osteotomy site and fixed segments in all patients. Neurological function improved from ASIA scale D to E in 5 patients. The patients were followed up for 30.4 months on average.

**Conclusion:**

Vertebral column decancellation is an effective treatment option for severe Pott’s kyphosis. The surgical planning software Surgimap Spine can be a reliable and helpful tool that provides a simplified method to evaluate and analyze the spino-pelvic parameters and simulate the osteotomy procedure. According to individual character, the appropriate surgery strategy should be selected.

## Background

Tuberculosis (TB) is an important cause of morbidity and mortality and The People’s Republic of China has the second highest incidence of TB worldwide [1]. Spinal TB is the most common form of extra-pulmonary TB, and accounts for nearly half of all musculoskeletal TB cases [2]. Delayed diagnosis is common and patients treated with anti-TB chemotherapy alone or with simple surgical debridement without fusion may develop disease reactivation [3]. In the late stage, bony destruction and vertebral collapse often leads to significant kyphosis, presenting clinically as a painful gibbus deformity, with increased instability, vertebral body translations and increased risk of neurologic involvement [4].

Anterior, posterior, and combined anterior and posterior (AP) procedures show various degrees of success for correcting kyphosis in TB [5–7]. The late correction of stiff and sharp angular deformities (more than 60°) is only feasible with three-column osteotomy or vertebral column resection (VCR) [8]. Pedicle subtraction osteotomy (PSO), 30–40°recommended as a safe range [9], is usually insufficient to correct severe kyphosis. Despite VCR is considered as the most powerful tool for correction of spinal deformity, this technique is a formidable last resort technique for severe fixed sagittal and coronal deformity due to its technical difficulty and potential for complications [10]. Vertebral column decancellation (VCD), a combination of the eggshell technique, Smith-Petersen osteotomy (SPO), PSO and VCR, is thought to be suitable for most patients with severe rigid kyphosis. VCD technique is a simpler and safer osteotomy procedure, while VCR provides the greatest amount of correction with higher risk of complications.

To obtain satisfactory outcomes, preoperative surgical planning must be performed carefully [11]. Several mathematical formulas [12–16] and graphical methods [17–20] for preoperative planning have been described. For most methods, however, global sagittal balance was not accurately evaluated and the effect of spinopelvic parameters, especially pelvic incidence (PI) and pelvic tilt (PT), were neglected, leading to postoperative undercorrection. Moreover, these are relatively complex for routine clinical use. An optimal approach to quantifying sagittal parameters and planning precise correction is lacking [21]. It is important to select the best surgery strategy in accordance with the characteristics of individuals. Surgimap Spine, a free computer program (Nemaris Inc., New York, NY, USA) that integrates spine-related measurement and tools for surgical planning in combination with knowledge gained from the published literature, offers a pragmatic graphical method for the surgical planning of osteotomies [21]. Surgimap Spine has been used in the surgical treatment of kyphosis deformity secondary to ankylosing spondylitis, [22] osteomalacia, or inappropriate past fusion procedures [23]. The software not only provides a practical and convenient method to analyze preoperative sagittal parameters and predict proper postoperative alignment, but also simulates the procedure of osteotomy to help surgeons select appropriate surgical methods.

In the current study, we evaluate the efficacy of VCD technique planned preoperatively with computer software-assistance, in the patients with Pott’s kyphosis.

## Methods

Twenty six patients diagnosed with Pott’s kyphosis were admitted to our department from May 2012 to May 2015. Diagnosis was based on radiographic examination, laboratory tests and histopathology. This study was conducted with approval from the Ethics Committee of Chinese PLA General Hospital and was performed in accordance with the Declaration of Helsinki. Written informed consent was obtained from all participants. The indications for surgery were as follows: (1) healed tubercular kyphosis; (2) low back pain refractory to conservative treatment; (3) inability to lie down in dorsal position; (4) increasing neurological deficit. Patients with active infection and who cannot tolerate surgery due to poor cardiopulmonary function were excluded. Surgical planning was performed using Surgimap Spine, development of an accurate plan required determination of parameters such as thoracic kyphosis (TK) and lumbar lordosis (LL). Patients whose fused vertebrae involved both T12 and L1, in which TK and LL cannot be measured, were also excluded.

**Fig. 1:**
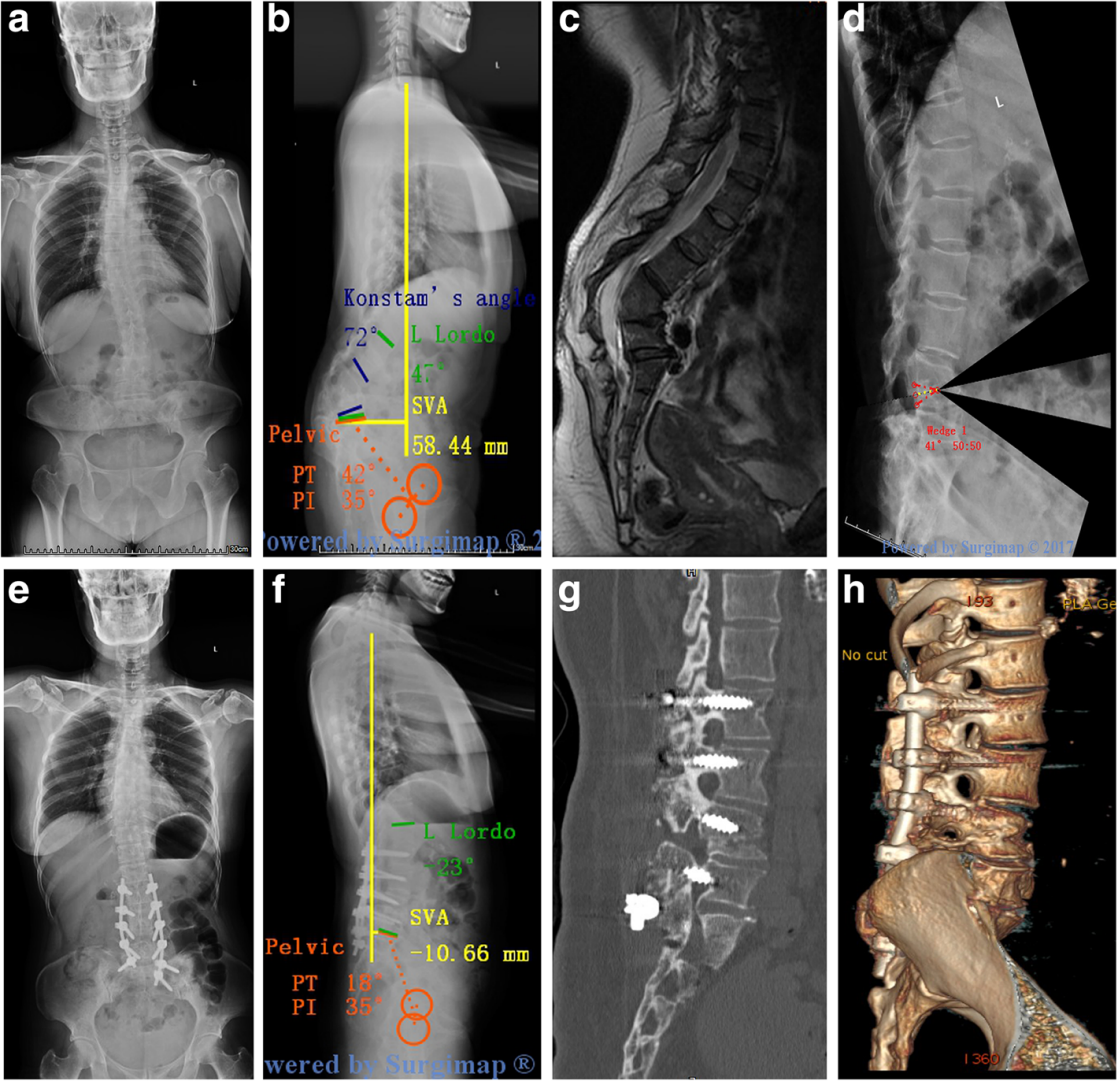
A 38-year-old man with Kyphotic deformity secondary to spinal Tuberculosis suffers from severe back pain and numbness in the left lower extremity. Pre-operative radiographs (**a**, **b**) and MRI (**c**) show that the apex of kyphosis is located at L3. L4, The Konstam’s angle was 72°, PI-LL: 82°, PT: 42°, The simulation of VCD osteotomy in Surgimap (**d**). The kyphosis was corrected to only 11° immediately after the surgery,PI-LL: 8°, PT: 18° (**e**, **f**, **g**). Three-dimensional reconstruction demonstrated that solid fusion of resection site was achieved 3.5 years postoperatively (**f**)

**Fig. 2:**
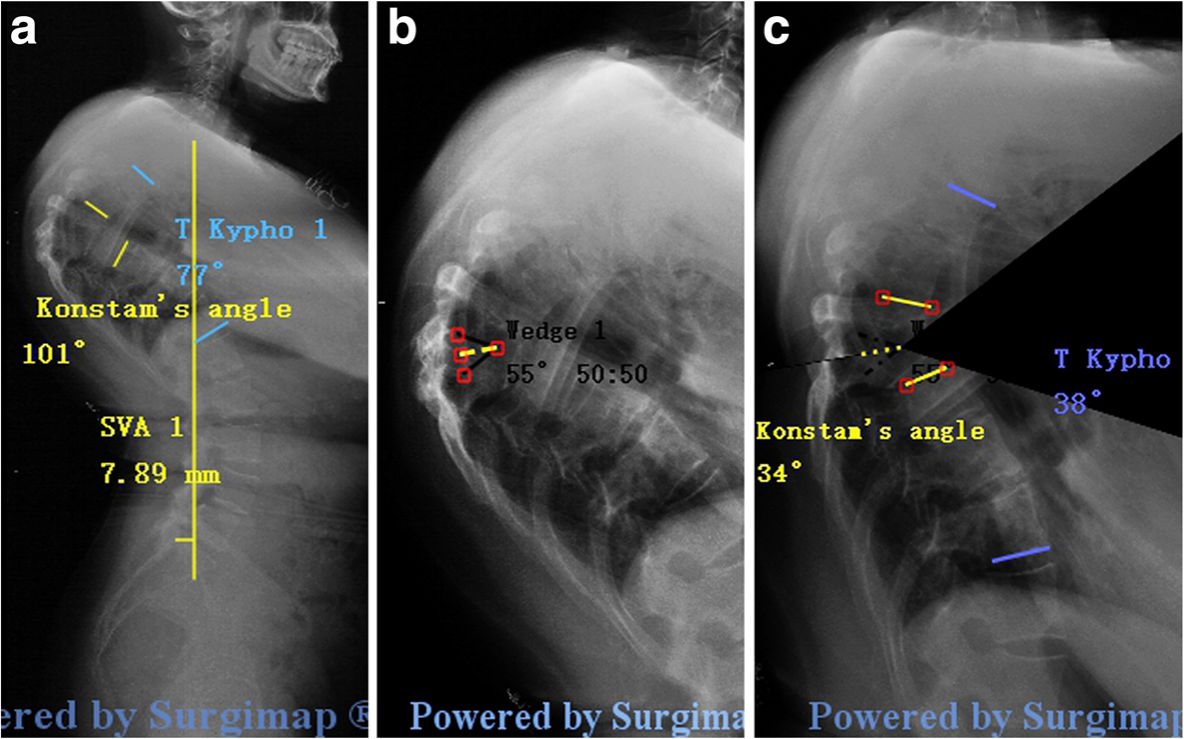
The process of Surgimap Spine for surgical planning. **a** Measurement the preoperative sagittal parameters: Konstam’s angle, SVA,TK; **b** Identification the site of osteotomy and the location of wedge-shaped resection. **c** Simulation of the osteotomy and evaluate the Surgimap-predicted postoperative parameters to check if VCD is sufficient for the correction

Anteroposterior and lateral spine radiographs, 3-dimensional computed tomography (3-D CT) reconstruction, and magnetic resonance imaging (MRI) were available for all patients (Fig. 1). The plain radiographs were taken in the standing position for all patients. One American Spinal Injury Association (ASIA) C patient (Case 7) had standing radiographs 2 weeks prior to admission. At that time, he had ASIA grade D and could stand unsupported. However, he declined surgery because of financial issues. However, his neurological deficits rapidly progressed and his ASIA grade deteriorated to C before surgery. Lateral radiography was processed with Surgimap Spine. Local and global sagittal parameters were measured and analyzed including Konstam’s angle [24], sagittal vertical angle (SVA),lumbar lordosis (LL), thoracic kyphosis(TK), pelvic tilt (PT), pelvic incidence (PI). Preoperative planning was executed in 3 phases [22]: 1. Spino- pelvic parameters were measured and analyzed (Fig. 2a). 2. Identification of the site of osteotomy and the location of wedge-shaped resection (Fig. 2b). The level of osteotomy wasselected with these fused vertebrae treated as one targeted vertebra. 3. Simulation of the osteotomy and evaluation of Surgimap-predicted postoperative parameters (Fig. 2c). This phase consists in applying a resection angle at the posterior column using the “Wedge Osteotomy” tool and graphically tracing the osteotomy directly on the radiographic image. The success of correction according to physiological limits was set at: SVA < 5 cm, TK < 50° (the apical vertebra was located at the thoracic level), PI-LL < 10° and PT < 20° [25] (the apical vertebra was located at the lumbar level). It was checked if VCD was sufficient for the correction. If not, VCR was applied. Of note, VCR, as the most aggressive method, allows a greater correction angle. However, it is a technically demanding and exhausting procedure with higher risk of major complications. It is generally the last-resort technique for severe spinal deformity [10].

Of 26 patients, 2 were excluded because of poor cardiopulmonary function and inability to tolerate surgery; 3 only had mild back pain without a neurological deficit, and refused surgery; and 3 were advised to have VCR surgery because their severe kyphosis was found to be inappropriate for VCD, based on osteotomy simulation using Surgimap. In all, 18 patients of whom (8males, 10 females; mean age 37.7 years) underwent the VCD technique. The mean Konstam’s angle was 88.1° (ranging from 70° to 105°). Among the 18 patients, 5 patients had previously underwent an initial debridement without fusion. Neurologic deficits were assessed according to the American Spinal Injury Association (ASIA) grading system as follows: ASIA E, 10 cases; ASIA D, 7 cases; and ASIA C, 1 case. Pain was assessed using the visual analogue scale (VAS). Disability status was assessed using the Oswestry Disability Index (ODI). All patients’ radiological and clinical records were recorded preoperatively, postoperatively and during the last follow-up period. Operation time, blood loss, and osteotomy levels were noted (Table 1).

**Table 1:** Demographic and clinical data

Patient	Age/Sex	ASIA	Osteotomy level	Instrumented levels	Operative time	Blood loss	Follow-up
					(min)	(ml)	(mon)
1	32/M	E	T8-T9	T6-T8,T10-T12	230	450	28
2	41/M	D	T9-T11	T7-T9,T12-L2	260	600	34
3	28/F	E	L2-L3	T12-L2,L4-S1	210	430	30
4	35/M	D	T9-T10	T7-T9,T11-L1	200	450	32
5	37/F	D	L1-L2	T11-L1,L3-L5	220	500	29
6	33/F	E	L1-L2	T11-L1,L3-L5	230	400	31
7	39/M	C	T8-T9	T6-T8,T10-T12	230	420	27
8	44/F	D	T10-T12	T8-T10,L1-L3	220	480	26
9	29/M	E	L1-L2	T11-L1,L3-L5	240	500	29
10	40/F	D	T10-T12	T8-T10,L1-L3	260	550	30
11	36/M	E	L2-L3	T12-L2,L4-S1	200	430	35
12	44/F	D	T10-T12	T8-T10,L1-L3	220	500	31
13	43/F	E	T11-T12	T8-T11,L1-L3	240	580	36
14	35/F	E	T9-T10	T6-T9,T11-L1	210	460	29
15	46/F	D	T11-T12	T9-T11,L1-L3	220	420	28
16	38/M	E	L3-L4	L1-L3,L5-S1	240	500	34
17	42/M	E	L1-L2	T11-L1,L2-L4	200	430	31
18	36/F	E	T8-T9	T6-T8,T10-T12	220	450	28

*ASIA* indicates American Spinal Injury Association

### Operative technique

All surgeries were performed under monitoring of somatosensory-evoked potentials, transcranial motor-evoked potentials, and free-running electromyography. Under general anesthesia, the patient was placed prone on the operating table, and a standard posterior middle incision was made at the predetermined level. The spine was exposed by dissection lateral to the costotransverse joint at the thoracic level and the lumbar transverse process. The segmental vessels were coagulated using electric cauterization and hemostatic gauze. Pedicle screws (Weigao Orthopedic,Shandong, China) were then placed three levels above and below the damaged vertebral body by freehand technique. C-arm fluoroscopy was used to confirm the appropriate insertions.

**Fig. 3:**
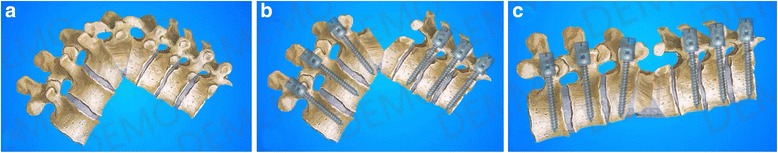
VCD osteotomy for Pott’s deformity. **a** Pott’s Kyphotic deformity **b** A wedge-shaped posterior column was removed **c** The anterior column was opening when the posterior wedge space was closed

Then, VCD was performed (Fig. 3). A pedicle probe and drill were used to create and enlarge relatively normal pedicle holes in the target vertebra on both sides of the pedicles. Cancellous bone of the posterior half of the osteotomy column was adequately removed through the pedicle holes, using a rongeur and curette. A high-speed drill was used to thin the anterior cortex and lateral walls of the vertebral body and linear fractures of the anterior cortex were created using an osteotome. Then the spinal canal was opened laterally, and the posterior elements including the spinous process, bilateral lamina, transverse process, and the adjacent facet joints were removed. After removing the posterior cortical bone of the osteotomized vertebra, the kyphotic spine was corrected using gentle manual force,and was stabilized with a temporary rod. The operating table and position of the patient were adjusted for correction.

**Fig. 4:**
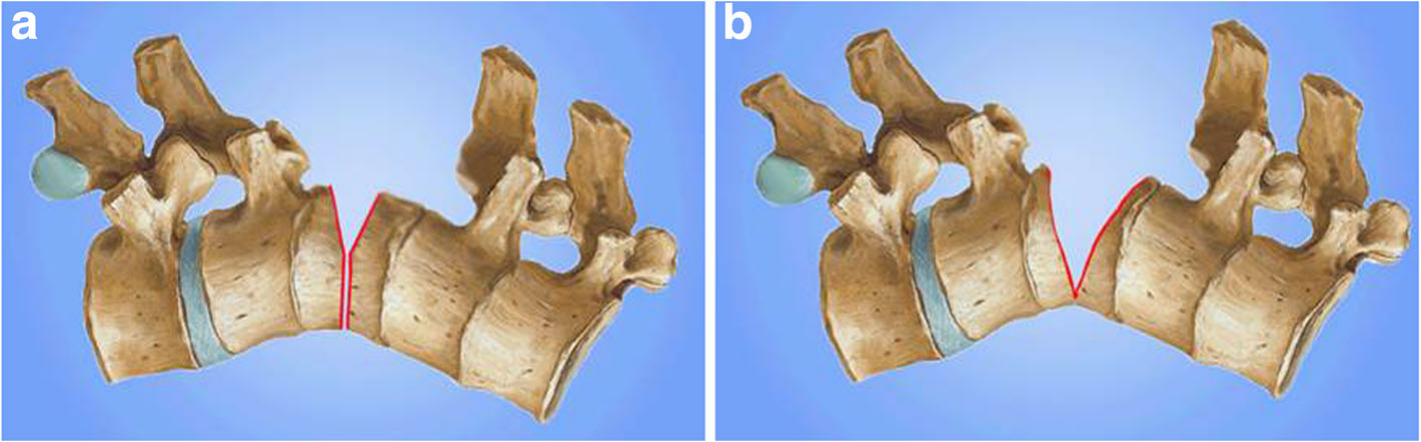
Comparison of VCD and PSO. **a** Vertebral column decancellation is a “Y” type osteotomy **b** Pedicle subtraction osteotomy is a “V” type osteotomy

During the correction procedure, an anterior opening wedge was created and the hinge was located at the border of the anterior and medial column. VCD technique is a “Y” type osteotomy rather than a “V” type osteotomy (PSO) (Fig. 4). The posterior interlaminar fusion was completed over the fixed segments with residual autogenous bone. After confirmation of absent soft or bony compression, a drainage tube was placed in the surgical field, and the wound was closed in layers.

### Statistical analysis

All statistical analysis was performed with SPSS v19.0 software (SPSS Inc., Chicago, Illinois). Paired t tests were used to determine significant changes between data that were collected preoperatively and postoperatively; the Wilcoxon signed rank test was used as a nonparametric alternative for which the required conditions were not satisfied, and the one-way ANOVA test was performed to compare mean values of three samples. *p*-value < 0.05 was considered statistically significant.

## Result

VCD osteotomy was performed in all patients according to preoperative surgical planning. All patients completed follow-up of 30.4 months on average, ranging from 26 to 36 months. The average operation time was 225 min (range, 200–260 min) with a mean intraoperative blood loss of 475 mL (range, 400–600 mL). The Konstam’s angles decreased from 88.1° (range, 70–105°) preoperatively to 18.5° (range, 7–31°) at the final follow-up (*P* < 0.01). All patients reached the physiological limits and the preoperative and last follow-up data for the 18 patients are shown in Table 2.

**Table 2:** The preoperative and last follow-up data

Parameters	Pre-operative	Post-operative	*p*-value
Konstam’s angle(°)	88.1	18.5	<0.001
TK(°)^a^	76.9	31.1	<0.001
PI-LL(°)^b^	78.7	5.7	<0.001
PT(°)^b^	43	14.3	<0.001
SVA(cm)	5.3	2.4	<0.001
VAS	7.1	1.8	<0.001
ODI(%)	65.8	20.2	<0.001

*TK* thoracic kyphosis, *PI* pelvic incidence, *LL* lumbar lordosis, *PT* pelvic tilt, *SVA* sagittal vertical angle, *VAS* visual analogue score, *ODI* oswestry disability index

^a^*n* = 11, measured when the apical vertebra was located at the thoracic level;^b^*n* = 7, measured when the apical vertebra was located at the lumbar level

The mean VAS score decreased from a preoperative 7.1 (range, 6–8) to 1.8 (range, 1–3, *P* < 0.01) and the ODI improved from 65.8% (range, 58–74%) to 20.2% (range, 12–38%, *P* < 0.01). At final follow-up, there was radiographic evidence of solid fusion at the osteotomy site and fixed segments in all patients. Neurological function improved from ASIA scale D to E in 5 patients.

Dural tears were encountered in two cases that underwent an initial debridement, and were successfully repaired with 6–0 Prolene suture. No deep wound infection was identified. One patient had transient partial neurological deficit post-operatively and recovered completely within 3 months. To make a comprehensive comparison between VCD and VCR and PSO, we retrospectively reviewed 51 patients with Pott’s kyphosis who underwent three-column osteotomies (PSO, *n* = 23; VCD, *n* = 18, VCR, *n* = 10) between May 2010 and May 2015 in our department by 2 spinal deformity surgeons. All the patients completed a minimum of 2 years of follow-up. The data comparing radiological and clinical outcomes in the PSO, VCD, and VCR groups are shown in Table 3.

**Table 3:** Comparison of the PSO‚ VCD‚ VCR group in radiological and clinical outcomes

	VCD	PSO	VCR	*p*-value
Patients	18(8 M,13F)	23(12 M,11F)	10(6 M, 4F)	
Mean age (yrs) (range)	37.7(29 to 46)	35.6(25 to 43)	41.2(31 to 48)	0.23
Preoperative Konstam’s angle(°)	88.1(70–105)	63.5(52–81)	96.4(82–131)	0.04
Follow-up Konstam’s angle(°)	18.5(7–31)^&^	16.3(8–29)^&^	32.1(18–42)^&^	0.02
Preoperative SVA(cm)	5.3(3.6–5.9))	4.5(2.2–5.1)	5.7(3.3–6.2)	0.31
Follow-up SVA(cm)	2.4(1.2–4.1)^&^	1.5(1.2–4.1)^&^	3.4(2.2–5.1)^&^	0.27
Preoperative TK(°)*	76.9(53–96)	65.2(51–85)	84.8(73–113)	0.03
Follow-up TK(°)*	14.3(10–23)^&^	16.3(12–32)^&^	32.5(24–62)^&^	0.02
Preoperative PI-LL(°)^#^	78.7(53–89)	62.3(42–74)	83.5(65–96)	0.01
Follow-up PI-LL(°)^#^	5.7(3–9)^&^	6.1(2–10)^&^	9.4(5–17)^&^	0.01
Operating time (mins)	225(200–260)	237(200–280)	370(320–400)	0.03
Loss of blood (ml)	475(400–600)	420(380–600)	625(500–980)	0.02
Complications (n)(%)	3(16.7)	4(17.4)	3(30)	0.03
Time of hospitalization(d)	11.3(7–17)	10.5(7–15)	15.2(10–21)	0.43
Preoperative VAS	7.1(6–9)	7.3(6–9)	8.4(7–9)	0.12
Follow-up VAS	1.8(1–3)^&^	1.5(1–4)^&^	4.1(3–7)^&^	0.02
Preoperative ODI(%)	65.8(59–84)	63.6(57–81)	74.8(65–89)	0.24
Follow-up ODI(%)	20.2(15–41)^&^	21.1(16–38)^&^	35.6(25–51)^&^	0.03

*VCD* vertebral column decancellation, *PSO* pedicle subtraction osteotomy, *VCR* vertebral column resection, *TK* thoracic kyphosis, *PI* pelvic incidence, *LL* lumbar lordosis, *SVA* sagittal vertical angle, *VAS* visual analogue score, *ODI* oswestry disability index. ^&^< 0.05 vs. preoperative data; *n = 11, measured when the apical vertebra was located at the thoracic level ; ^#^*n* = 7,measured when the apical vertebra was located at the lumbar level.

## Discussion

TB spondylitis can lead to significant osteolysis and collapse of the vertebral bodies, resulting in hyperkyphosis and tethering of the spinal cord [26]. Late stages of rigid hyperkyphosis are difficult to treat [8]. The sharp angular hyperkyphosis often requires complex three-column osteotomies. Currently, the one-stage posterior approach is most often used for minimizing the risk of injury to anterior vascular and visceral structures. PSO, the most popular osteotomy technique, has been applied for progressive TB thoracic and thoracolumbar kyphosis. The osteotomy should be limited to 30–40° as a safe single segment range; otherwise, the spinal cord is excessively shortened and distorted [27]. Although VCR or its variation, posterior-only VCR, can provide the greatest amount of surgical correction when compared to all other spinal osteotomy types [28], its use is limited due to its high inherent neurological risk related to instability induced during correction of the malformation [29, 30]. The complication rate has been estimated as high as 59% for posterior VCR [31].

Therefore, we prefer the VCD technique. This technique allows greater correction angles than PSO, and is a simpler and safer procedure with lower risk of complications compared with VCR. The main challenge for VCD is in determining the exact location of the hinge intraoperatively. The location of the hinge differs and the postoperative correction angle is variable. Until now, the surgeon’s experience was the determinant. To solve this challenge, Surgimap was used for preoperative planning and C-arm technique was used to confirm the appropriate position. VCD is a newer technique, with insufficient data on long-term outcomes for assessment of effectiveness. As our medical team follows up all patients, more long-term outcomes of VCD technique for spinal deformity will be reported.

**Fig. 5:**
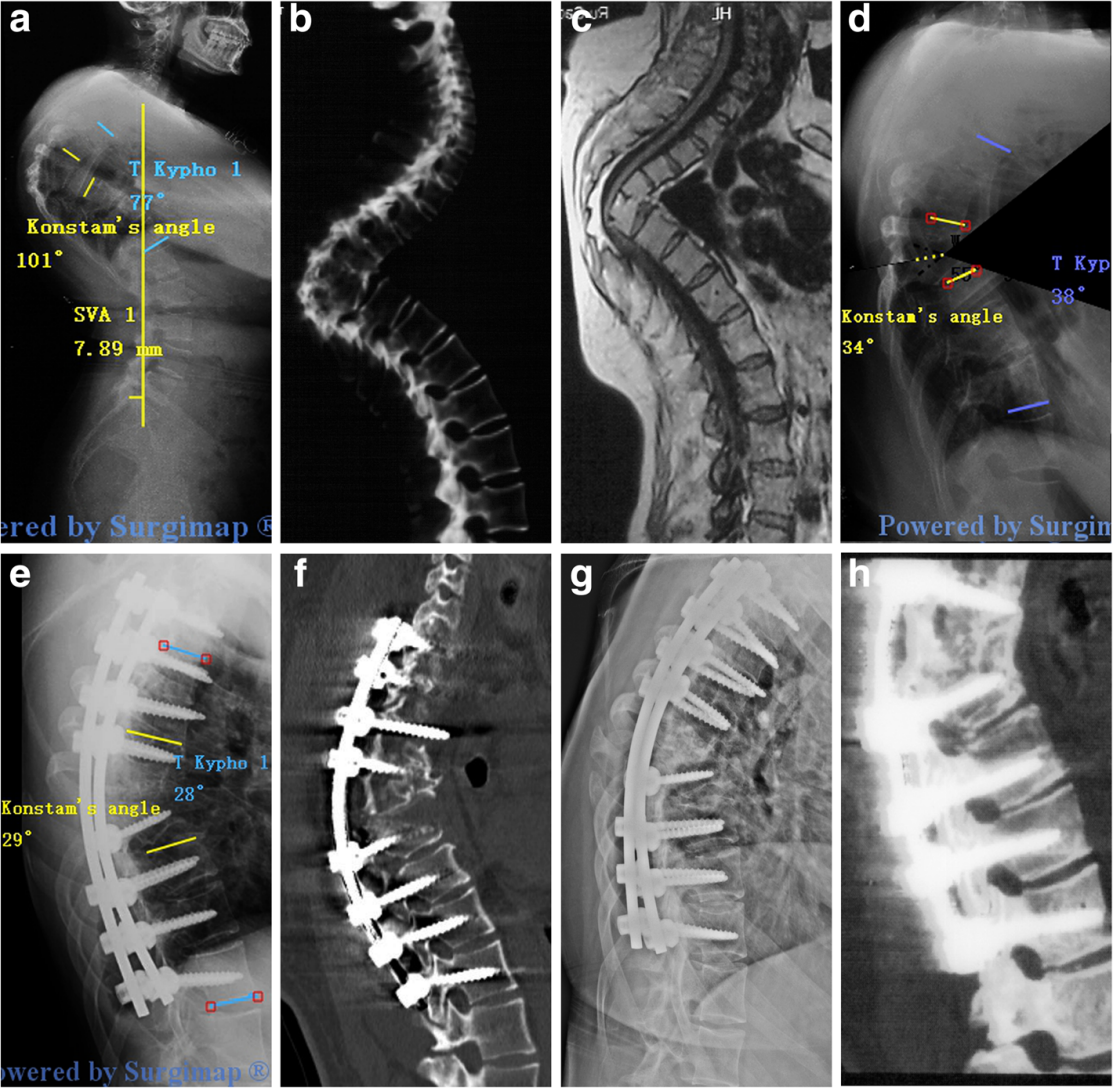
A 36-year-old patient with Pott’s deformity. The patient’s main complaints were increasing neurological deficit and cosmetic issues. Pre-operative radiographs (**a**), CT (**b**) and MRI (**c**) show that the apex of kyphosis is located at T6-T9, The Konstam’s angle was 101° and the TK was 77°. The simulation of VCD osteotomy in Surgimap (**d**). The Konstam’s angle was corrected to 29° and the TK was 28° immediately after the surgery (**e**, **f**). 28 months follow-up X-ray (**g**) and CT (**h**) scan show a solid fusion

Of note, no way is perfect. VCD is not suitable for all deformities. Our maximum preoperative Konstam’s angle was 105°. VCR, as the most aggressive method, is still recommended for some severe kyphosis despite of the high risk of complications. Thus, it is important to select the appropriate individual treatment. The surgeon-developed surgical planning software, Surgimap Spine, is increasingly popular among spine surgeons. It offers a pragmatic and systematic approach involving evaluation of important spinopelvic parameters and simulation of spinal osteotomy with the goal of optimal treatment. As VCD technique is a “Y” type osteotomy, the Wedge Osteotomy tool was used in surgical planning using Surgimap. The portion of the wedge-shaped resection was determined by two lines consisting of three points. The anterior point was located at the border of the anterior and medial column. The cephalad and caudad junction points of the pedicle and vertebral body were selected as the posterior points of the upper and lower resection line. The wedge shape was set to a 50:50 angle bisector. Upon application of the osteotomy, the image was modified directly: the wedge shape portion was removed, the posterior column closed, and the anterior column opened (Fig. 1d and Fig. 5d). Then, the key parameters were measured to determine whether sagittal alignment was restored to the normal physiological limits. Subsequently, the optimal surgical strategy was decided. Intraoperatively, it was essential to determine the anterior point of the wedge-shaped resection. Generally, the length between the anterior point and the pedicle is measured in Surgimap; after placing the pedicle screw above and below the damaged vertebral body, a pedicle probe with appropriate depth was inserted into the targeted vertebral body through the pedicle hole. C-arm fluoroscopy confirmed the exact location of the probe and the anterior point was determined. A high-speed drill was used to enlarge the pedicle hole both cephalad and caudad, until the corresponding walls were penetrated. The three points of the wedge-shaped resection were determined intraoperatively. Good surgical planning requires exact and full evaluation of sagittal alignment. Surgimap not only measures global and local key parameters, but also takes compensatory mechanisms used in an effort to maintain the trunk and the effect of spinopelvic parameters into account. As a geometrical method, Surgimap allows the surgeon to introduce given angular corrections at any given point of the spine and mimic the surgical maneuvers on preoperative X-rays, thus providing an estimate of the postoperative results. Its novel approach provides a virtual preview of the result of a surgical procedure, and allows the surgeon to evaluate the amount of correction needed and to determine its potential effect. Moreover, Surgimap, an open source archive system and software, is very easy to learn and convenient to use. It is an effective and practical tool for spine surgeons. In 2014, Yunus Atici et al. [23] used Surgimap Spine to evaluate the efficacy of two-level PSO osteotomy, and concluded that computer software (Surgimap) seems to fail as an assisting method in the preoperative surgical planning of sagittal plane deformities, since its incapability of estimation the amount of bleeding and the possible buckling of the spinal cord. In my opinion, however, The main purpose of surgical planning for a spine deformity is to analyze the coronal and sagittal alignment and evaluate the effects of predicted surgical technique in order to choose the best procedure for a given patient. In the current study, Surgimap was used to measure pre- and postoperative spinal parameters and to simulate an osteotomy procedure. The geometrical changes of spinopelvic parameters help surgeons select the optimal osteotomy. It’s very helpful and useful method regarding the aspect. The amount of bleeding and spinal cord buckling are influenced by a number of factors, including patient coagulation function, duration of the operation, and the surgeon’s experience and skill. Thus, it is difficult to predict outcomes with any existing method and further research is needed. The other weak point of the software is that it is not possible to consider the reciprocal changes that may occur in the unfused segment [21]. In the current study, we found that all patients achieved the satisfactory photographic clinical outcomes as planned before surgery. The reason may be that the main problem in patients with TB kyphosis is the imbalance in local parameters and the compensatory change of unfused segments in the direction of global sagittal balance. When using Surgimap Spine, surgeons should be aware of the following points: first, all angle and length parameters must be measured precisely, because a small error has a large effect on the outcome. Second, before measuring any length, image calibration must be performed. The surgeon draws a line on the image and then defines the realistic length of the line. Third, it is important to make sure that preoperative planning is consistent with surgery, so X-ray or CT should be performed intraoperatively to verify that the location of osteotomy is as planed with Surgimap.

## Conclusion

VCD is an effective treatment option for severe Pott’s kyphosis. The Surgimap Spine surgical planning software can be a reliable and helpful tool that provides a simplified method to evaluate and analyze the spino-pelvic parameters and simulate the osteotomy procedure. The appropriate surgical strategy should be selected according to individual findings.

## Abbreviations

AP: Anterior and posterior
LL: Lumbar lordosis
MRI: Magnetic Resonance Imaging
ODI: Oswestry Disability Index
PI: Pelvic incidence
PSO: Pedicle subtraction osteotomy
PT: Pelvic tilt
SPO: Smith-Petersen osteotomy
SVA: Sagittal vertical angle
TK: Thoracic kyphosis
VAS: Visual analogue score
VCD: Vertebral column decancellation
VCR: Vertebral column resection
